# Characterizing the Interaction between Root-knot Nematodes and Soil-borne Fungi which are Pathogenic to Passion Fruit (*Passiflora Edulis*)

**DOI:** 10.2478/jofnem-2022-0023

**Published:** 2022-07-20

**Authors:** Mariana Z. Mangeiro, Rafael A. Nunes, José O.L. Vieira, Vicente Mussi-Dias, Alexandre P. Viana, Ricardo M. Souza

**Affiliations:** 1 Department of Entomology and Plant Pathology, Universidade Estadual do Norte Fluminense Darcy Ribeiro (UENF), Campos dos Goytacazes, Brazil; 2 Department of Agricultural Engineering, UENF, Brazil; 3 Department of Plant Breeding, UENF, Brazil

**Keywords:** disease complex, *Fusarium*, interaction, *Meloidogyne incognita*, *Meloidogyne javanica*, *Neocosmospora*, *Passiflora edulis*, passion fruit, root-knot nematodes, soilborne fungi, synergistic interaction

## Abstract

For decades there have been anecdotal claims of synergistic interactions between plant-parasitic nematodes and soil-borne fungi causing decline of productivity of passion fruit (*Passiflora edulis*) orchards. An empirical confirmation of these disease complexes would impact disease management and plant breeding for resistance. To test those claims, we subjected passion fruit plants to single or concomitant parasitism by *Meloidogyne javanica* or *M. incognita* and *Fusarium nirenbergiae* or *Neocosmospora* sp. under controlled conditions. Non-inoculated plants served as control for the assays. The severity of shoot symptoms and variables related to plant growth, the extent of fungal lesions, and nematode reproduction were assessed to characterize the interactions. The shoot symptoms and effect on plant growth induced by the pathogens varied, but no synergy between the pathogens was observed. Moreover, the volume of tissue lesioned by the fungi was not affected by co-parasitism of the nematodes. Conversely, plant resistance to the nematodes was not affected by co-parasitism of the fungi. The interactions *M. incognita*-*F. nirenbergiae*, *M. incognita-Neocosmospora* sp., *M. javanica-F*. *nirenbergiae*, and *M. javanica-Neocosmospora* sp. were not synergistic as previously claimed, but instead neutral.

Passion fruit (*Passiflora edulis* Sims) stands out among the exotic tropical fruits with a global production of about 1.4 million tons/year (Altendorf, 2018). The fruit´s global export value reached 2.7 billion dollars in 2020, due to increasing demand by the United States and European countries (Tridge, 2021).

Unfortunately, several plant diseases have forced many passion fruit growers out of this market. For instance, in Brazil, which is responsible for two-thirds of world production of passion fruit (Altendorf, 2018), the area cultivated with passion fruit decreased by one-third in the last decade (Instituto Brasileiro de Geografia e Estatística, 2021). Typically, a passion fruit orchard provides three or four harvests before needing renewal. However, in many regions which produce passion fruit, orchards now provide just one harvest before declining due to plant diseases (José and Pires, 2011).

Key pathogens of passion fruit are the reniform nematode *Rotylenchulus reniformis* Linford and Oliveira; © the root-knot nematodes *Meloidogyne javanica* (Treub) Chitwood and *M. incognita* (Kofoid and White) Chitwood; and the soil-borne fungi *Fusarium nirenbergiae* L. Lombard and Crous (= *F. oxysporum* f.sp. *passiflorae*) and *Neocosmospora* sp. (= *F. solani* f.sp. *passiflorae*, *sensu* Sandoval-Denis *et al*., 2019). These pathogens occur in passion-fruit-producing regions worldwide.

Root-knot and reniform nematodes cause chlorosis and wilting of leaves, stunted growth, and poor root system, which may present rotted rootlets. Seedlings and mature plants may succumb to heavy parasitism (Silveira and Araújo, 2011). *F. nirenbergiae* and *Neocosmospora* sp. cause fusarium wilt and collar rot, respectively, with the following similar symptoms: chlorosis and wilting of leaves; cracking of stem bark; lesions in the roots, collar region, and vascular tissues; and death (Laranjeira *et al*., 2018).

There have been claims of synergistic interactions between nematodes and soil-borne fungi in passion fruit. In synergistic interactions – often referred to as disease complexes – the combined effects of the pathogens are greater than the sum of the effects of each pathogen alone (Evans and Haydock, 1993; Francl and Wheeler, 1993). Based on field observations in Fiji, Kirby (1978) suggested that *R. reniformis* could be involved in the decline in the productivity of passion fruit orchards, which was initially attributed only to *Phytophthora cinnamomi* Rands and *P. nicotianae* Breda de Haan f.sp. *parasitica*. Field observations in Brunei, Brazil, and Venezuela also led to suggestions that *R. reniformis* increases the severity of *P. nicotianae* f.sp. *parasitica*, *F. nirenbergiae*, and *Fusarium* sp., but no data were provided (Peregrine and Yunton, 1980; Liberato, 2002; Castellano *et al*., 2012). Synergy between pathogens has been mentioned but was never properly investigated for over 40 years.

However, there have been two attempts to investigate such interactions. *Neocosmospora* sp. induced slightly longer lesions in the collar region of plants co-parasitized by *M. incognita*, in comparison with plants parasitized by the fungus only (Fischer *et al*., 2010). However, the authors assessed just one variable and employed few plant replicates/ treatments along with no assay replication. Rocha *et al*. (2021) were unable to obtain a consistent fungal infection of passion fruit plants, which precluded statistical analysis to infer pathogen interactions.

Disease complexes involving soil-borne fungi and nematodes have been reported in several other crops (Evans and Haydock, 1993; Francl and Wheeler, 1993; Back *et al*., 2002; Manzanilla-López and Starr, 2009). In most cases, *Fusarium*, *Phytophthora*, and *Meloidogyne* are the genera involved. Their synergy may give rise to new diseases; worsening of symptoms induced by either pathogen alone; reduction of plant tolerance to either pathogen; or poor growth of a cultivar known to be resistant to one of the pathogens, which is often referred to as “resistance breaking”. Soil-borne fungi and nematodes are intractable on their own, and disease complexes pose further hurdles to crop management. For instance, major losses are caused by disease complexes to soybean in the US, coffee in Central America, guava in Brazil and cotton worldwide (Gomes *et al*., 2011; Villain *et al*., 2013; Sanogo and Zhang, 2015; Navi and Yang, 2016).

We hypothesized that *M. incognita*, *M. javanica*, and *R. reniformis* interact synergistically with *F. nirenbergiae* and *Neocosmospora* sp., worsening plant damage. Corroboration of this hypothesis would underscore the importance of phytosanitary measures such as assessing the presence (or not) of root-knot and reniform nematodes when selecting a new cultivation area. Moreover, breeding programs would need to develop cultivars and rootstocks resistant to soil-borne fungi and nematodes to maximize performance in fields infested with both groups of pathogens.

To test our hypothesis, we subjected passion fruit plants to singular or concomitant parasitism by the nematodes *M. javanica*, *M. incognita* or *R. reniformis*, and the fungi *Neocosmospora* sp. or *F. nirenbergiae*, under controlled conditions. A lapse between inoculations of nematodes and fungi was employed to allow the former to interfere in the plants’ physiology, a tenet in disease complexes (Manzanilla-López and Starr, 2009). Noninoculated plants served as controls. Shoot symptoms and several variables were assessed to characterize the pathogens’ interactions. This article reports the interactions between *M. javanica* and *M. incognita* with those fungi. The *R. reniformis*-fungi interactions will be reported elsewhere.

## Material and Methods

### Assay procedures

**Figure S1:**
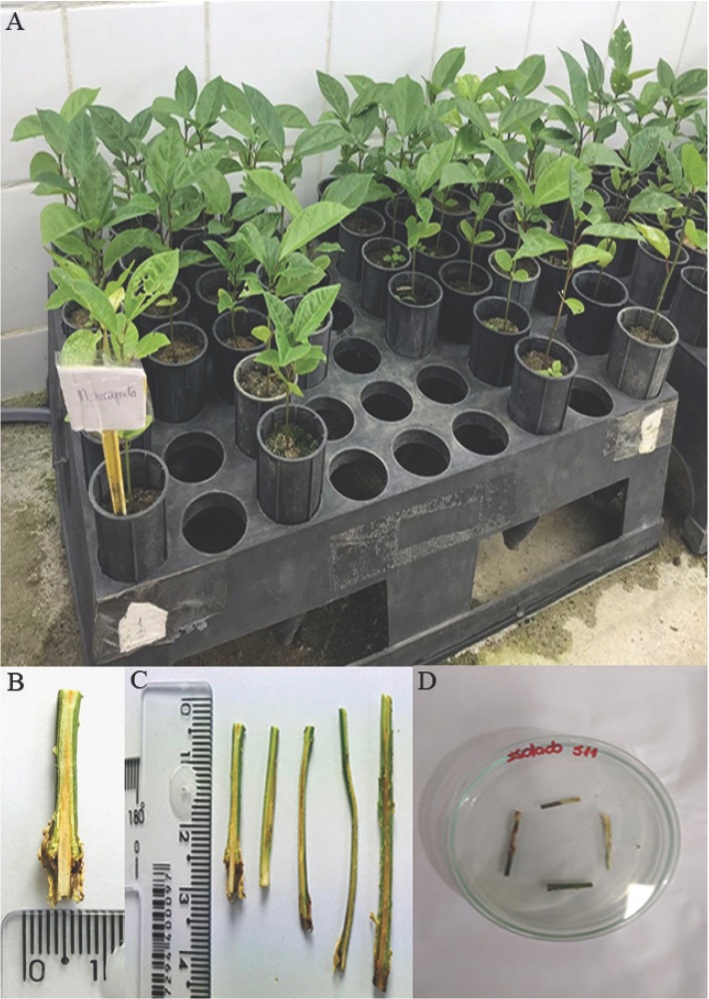
(A) Aspect of passion fruit plants in growth chamber assay to investigate fungusnematode interactions; (B) width of collar lesion caused by *Fusarium nirenbergiae* F022; (C) length of collar lesions caused by *F. nirenbergiae* F022 (two stems on the left) and *Neocosmospora* sp.; (D) fungus re-isolation from lesioned tissues.

Seeds of passion fruit cultivar UENF Rio Dourado were purchased from Rio Norte Sementes (Campos, Brazil) and sown in 300-cm^3^ plant growth tubes (Supplementary Fig. 1A) filled with a substrate composed of washed riverbed sand and soil (2:1). The substrate was subjected to solarization in tubular metal containers (12-cm diameter) for 3 d before use. The temperatures inside the containers (that is, 60–80^o^C) are known to eradicate both fungi and nematodes (Martins *et al*., 2003). The plants were cultivated in a growth chamber under a 12-hr photoperiod, with illumination of 106 m × mol^-1^ × m^-2^ provided by 40-watt fluorescent light tubes. The day and night temperatures were set to 27 and 26^o^C, respectively.

The plants were assigned to one of the following treatments: (i) inoculation with the nematode 60 d after seeding (DAS); (ii) inoculation with the nematode at 60 DAS and fungus at 120 DAS; or (iii) inoculation with the fungus at 120 DAS. Noninoculated plants served as controls. The treatments were randomly arranged in trays, with 10 replicates/treatment, for a total of 40 plants. This experimental design was employed to investigate the interactions of *M. incognita*-*Neocosmospora* sp. (isolates 311 and 511); *M. incognita*-*F. nirenbergiae* (isolate 022); *M. javanica*-*Neocosmospora* sp. (311 and 511); and *M. javanica*-*F. nirenbergiae* (022). For each interaction, two identical and independent assays (1 and 2) were conducted.

*M. incognita* and *M. javanica* were obtained from cultures maintained in tomato (*Solanum lycopersicum* L.) plants in a greenhouse at Universidade Federal de Viçosa (Brazil). Their identity was ascertained by morphology and esterase phenotyping (Esbenshade and Triantaphyllou_,_ 1985). These cultures were transferred to and maintained in passion fruit plants in a greenhouse. To obtain nematode inocula, infected roots were cleaned with tap water and submitted to nematode extraction through the method of Coolen and D’Herde (1972) but without adding kaolin in the blender. The resulting suspension was passed through 65- and 500-mesh sieves (250 and 25 mm openings, respectively). The nematode eggs and second-stage juveniles (J^2^) retained on the 500-mesh sieve were suspended in water, and three 1 mL aliquots were counted on Peter’s slides under a stereomicroscope. The inoculum was calibrated to 350 eggs + J^2^/mL of water. For inoculation, two 5-cm deep holes were made in the soil around the plant, into which 10 ml of the inoculum (3,500 eggs + J^2^) was applied. Ten tomato seedlings “Santa Cruz”, known to be susceptible to root-knot nematodes, were inoculated using the same procedures to attest to the viability of the inocula. These plants were cultivated under the same condition as the passion fruit, except in 5 L plastic pots.

*Neocosmospora* sp. 311, 511, and *F. nirenbergiae* 022 originated from passion fruit orchards which were affected by collar rot and fusarium wilt in the municipalities of Campos dos Goytacazes, Viçosa, and Cáceres (Brazil), respectively. Stock cultures maintained in anhydrous silica gel were routinely revived, inoculated in passion fruit plants, cultivated in a growth chamber, and re-isolated to avoid losing pathogenicity. For inoculum preparation, the fungi were cultivated in potato dextrose agar (PDA) medium in Petri dishes for 7 d, at 24^o^C, and in a 12-hr light/ dark cycle. Inoculation was performed according to the method of Fischer *et al*. (2010): a cleft (10-mm long, 5-mm wide, 1-mm deep) was made in the plant stem with a sterile blade, at 1 cm above the soil. A 6-mm wide agar disk collected from the edge of the fungus colony was placed on the exposed stem region and sealed with a sterile plastic film.

All assays were conducted until 165 DAS, except for those involving *Neocosmospora* sp. 311. This isolate always induced rapid decline of the plants, forcing evaluations at 135 DAS due to plant death. At the end of the assays, the plants were removed from the growth tubes, and the root system was gently cleaned with tap water. Nematode and fungal pathogenicity was expressed according to (i) the severity of shoot symptoms: leaf chlorosis, wilting, and leaf chlorosis, wilting and necrosis; and whole plant wilting and death (these symptoms were photographed and ranked on the modified scale of Santos (1997) – 1: no symptoms; 2: chlorosis restricted to one or two leaves; 3: leaf chlorosis in the whole plant and some leaf wilting; 4: whole plant wilting and necrosis of leaves; and 5: plant death); (ii) variables related to plant growth: plant length (PL) (from apical meristem to the most distal rootlet, in mm); shoot length (SL) (in mm); shoot fresh mass (SM) (in g); root system fresh mass (RSM) (in g) and root system volume (RSV) (calculated through water displacement in a graduated glass tube (in mm^3^); and (iii) the volume of lesioned tissue (VLT) in the collar-stem region (in mm^3^). Because fungi-induced lesions in the stem typically ascended uniformly in a sagittal plane, the length and width of the lesions were measured with a digital pachymeter (Supplementary Fig. 1B,C) and applied in the formula to calculate the volume of a cylinder – π × r^2^ × h – in which r is the radius and h is the height. The fungal damage was also expressed by the length of the lesion (LL).

Nematode reproduction was expressed as the final population (Pf), used in turn to compute the reproduction factor (RF = Pf/Pi). To obtain Pf, eggs and J_2_ were extracted from roots through trituration in a commercial blender (method of Boneti and Ferraz, 1981). The resulting suspension were sieved and counted as described before. This procedure was also employed in the tomato plants which were used to attest to the viability of the inoculum.

To confirm re-isolation of the fungi, fragments from inner tissues were collected in the boundary between lesioned and healthy tissues in the collar-stem region. These tissues were then disinfested in a 70% aqueous ethanol solution for 1 min and in a 0.5% aqueous sodium hypochlorite solution for 1 min. After double cleansing in sterile distilled water, the fragments were transferred to PDA medium and cultivated as described before. Seven days later, the colonies (Supplementary Fig. 1D) were identified to confirm the re-isolation of *Neocosmospora* sp. and *F. nirenbergiae*.

## Data availability and statistical analysis

The dataset of this article is available in Figshare at https://doi.org/10.6084/m9.figshare.14706870. For each nematode-fungus interaction, plant growth data from assays 1 and 2 were submitted to a normality test (Shapiro–Wilk) at 5% (Supplementary Table 1). With the exception of RSV, all variables showed normality of residuals. ANOVA was performed in which the explanatory variables were the different nematode and fungus treatments and the response variables were the quantitative variables assessed. When a significant (*P* < 0.05) difference was observed, the Tukey test at 5% was employed to compare the treatment means.

**Table 1:** Effect of pathogens, acting alone or combined, on the root system volume (in mm^3^) of passion fruits plants cultivated in replicated growth chamber assays (1 and 2).

Treatments	Interactions and assays	
	*Meloidogyne incognita-Neocosmospora sp. 311*	
	**1**	**2**
***Neocosmospora*** **sp. 311**	1500 a^a^	1400 b
***M. incognita***	1000 a	1700 b
***M. incognita-Neocosmospora*** **sp. 311**	1000 a	1400 b
**Uninoculated control**	1000 a	2900 a
	***M. incognita-Neocosmospora*** **sp. 511**	
	**1**	**2**
***Neocosmospora*** **sp. 511**	1800 a	1100 a
***M. incognita***	1000 b	1100 a
***M. incognita-Neocosmospora*** **sp. 511**	1000 b	1100 a
**Uninoculated control**	1200 b	1100 a
	***M. incognita-Fusarium nirenbergiae*** **F022**	
	**1**	**2**
***F. nirenbergiae*** **F022**	1000 a	1000 a
***M. incognita***	1400 a	1400 a
***M. incognita-F. nirenbergiae*** **F022**	1000 a	1000 a
**Uninoculated control**	1300 a	1200 a
	***Meloidogyne javanica-Neocosmospora*** **sp. 311**	
	**1**	**2**
***Neocosmospora*** **sp. 311**	1500b c	1000 a
***M. javanica***	2400 ab	1000 a
***M. javanica-Neocosmospora*** **sp. 311**	1400 bc	1000 a
**Uninoculated control**	2400 a	1000 a
	***M. javanica-Neocosmospora*** **sp. 511**	
	**1**	**2**
***Neocosmospora*** **sp. 511**	1800 a	1100 a
***M. javanica***	2000 a	1300 a
***M. javanica-Neocosmospora*** **sp. 511**	1000 b	1000 a
**Uninoculated control**	2400 a	1300 a
	***M. javanica-F. nirenbergiae*** **F022**	
	**1**	**2**
***F. nirenbergiae*** **F022**	2300 a	1000 a
***M. javanica***	2400 a	1000 a
***M. javanica-F. nirenbergiae*** **F022**	2100 a	1000 a
**Uninoculated control**	2600 a	1000 a

a In the column, different letters indicate significant difference between treatments according to the Kruskal–Wallis test at 5% significance. Values are mean of 10 replicates per treatment for each assay.

For RSV and the scale of symptom severity data could not be transformed appropriately. Hence, the analysis was performed using the nonparametric Kruskal–Wallis test.

The variables related to only one of the pathogens – such as nematode Pf and RF, VLT in the collar-stem region, and LL – were analyzed using the Shapiro–Wilk test at 5% (Supplementary Table 2). The variables Pf, RF, and VLT in the collar-stem region showed normality of residuals and were analyzed using the T-test at 5%. The variable LL showed normality of residuals in all but the interaction *M. javanica*-*F. nirenbergiae* F022. Because this would require different statistical treatments of the same variable, it was not further analyzed.

**Table 2:** Effect of the parasitism by *Meloidogyne incognita* or *M. javanica* on the volume of lesioned tissue in the collar-stem region (in mm^3^) caused by *Neocosmospora* sp. (311 or 511) or *Fusarium nirenbergiae* F022 in passion fruit plants cultivated in replicated growth chamber assays (1 and 2).

Treatments	Assays	
	**1**	**2**
***Neocosmospora*** **sp. 311**	62.78 a^a^	41.99 a
***M. incognita-Neocosmospora*** **sp. 311**	101.94 a	30.23 a
	**1**	**2**
***Neocosmospora*** **sp. 511**	82.64 a	36.04 a
***M. incognita-Neocosmospora*** **sp. 511**	72.24 a	38.03 a
	**1**	**2**
***F. nirenbergiae*** **F022**	167.58 a	20.77 a
***M. incognita-F. nirenbergiae*** **F022**	84.64 a	28.56 a
	**1**	**2**
***Neocosmospora*** **sp. 311**	162.42 a	47.79 a
***M. javanica-Neocosmospora*** **sp. 311**	95.55 a	33.13 a
	**1**	**2**
***Neocosmospora*** **sp. 511**	111.24 a	49.21 a
***M. javanica-Neocosmospora*** **sp. 511**	90.22 a	40.82 a
	**1**	**2**
***F. nirenbergiae*** **F022**	127.06 a	18.26 a
***M. javanica-F. nirenbergiae*** **F022**	125.41 a	29.76 a

a In the column, different letters indicate significant difference between treatments according to the *T* test at 5% significance. Values are means of 10 replicates per treatment for each assay.

All statistical analyses were performed using the software R (version 4.0.5), package “ExpDes”.

## Results

### Plant growth variables

The pathogens significantly affected variables expressing growth of passion fruit plants – SM, RSM, PL, and SL (Supplementary Table 3).

**Table 3:** Severity of shoot symptoms, ranked on a 1–5 scale, induced by pathogens acting alone or combined in passion fruits plants cultivated in replicated growth chamber assays (1 and 2).

Treatments	Interactions and assays		
	***Meloidogyne incognita*****-*Neocosmospora*** **sp. 311**	
	**1**	**2**
***Neocosmospora*** **sp. 311**	3.1 b^a^	3.5 b
***M. incognita***	0.9 a	1.4 a
***M. incognita***-***Neocosmospora*** **sp. 311**	3.3 b	3.4 b
Uninoculated control	1 a	1 a
	***M. incognita*****-*Neocosmospora* sp**. **511**	
	**1**	**2**
***Neocosmospora*** **sp. 511**	2 b	3.2 bc
***M. incognita***	2.3 b	3 b
***M. incognita***-***Neocosmospora*** **sp. 511**	2 b	3.8 c
**Uninoculated control**	1 a	1 a
	***M. incognita*****-*Fusarium nirenbergiae*** **F022**	
	**1**	**2**
***F. nirenbergiae*** **F022**	3.6 c	3 b
***M. incognita***	2.3 b	3 b
***M. incognita***-***F. nirenbergiae*** **F022**	2.7 bc	3 b
**Uninoculated control**	1 a	1 a
	***Meloidogyne javanica*****-*Neocosmospora*** **sp. 311**	
	**1**	**2**
***Neocosmospora*** **sp. 311**	3.7 c	3 b
***M. javanica***	2.6 b	3 b
***M. javanica***-***Neocosmospora*** **sp. 311**	3.3 bc	3.2 b
**Uninoculated control**	1 a	1 a
	***Meloidogyne javanica*****-*Neocosmospora*** **sp. 511**	
	**1**	**2**
***Neocosmospora*** **sp. 511**	2.4 b	3 b
***M. javanica***	2 b	3 b
***M. javanica***-***Neocosmospora*** **sp. 511**	2.1 b	3.2 b
**Uninoculated control**	1 a	1 a
	***M. javanica*** **-*F. nirenbergiae* F022**	
	**1**	**2**
***F. nirenbergiae*** **F022**	3.1 b	3 b
***M. javanica***	3 b	3 b
***M. javanica***-***F. nirenbergiae*** F022	3 b	3.3 b
**Uninoculated control**	2 a	1 a

a In the column, different letters indicate significant difference between treatments according to the Kruskal–Wallis test at 5% significance. Values are mean of 10 replicates per treatment for each assay.

**Figure 1:**
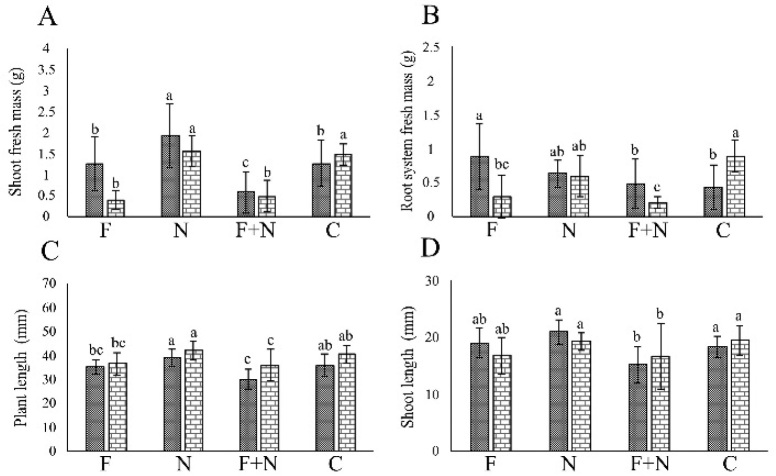
Effect of *Meloidogyne incognita* and *Neocosmospora* sp. 311, acting alone or combined, on the growth of passion fruit plants cultivated in a growth chamber, in the assays 1 (dotted column) and 2 (brick-pattern column). F: parasitism by fungus only; N: parasitism by nematode only; F + N: parasitism by fungus and nematode; C: noninoculated control. Columns with different letters indicate significant difference between treatments according to the Tukey test at 5%. Values are mean of 10 replicates per treatment for each assay.

**Figure 2:**
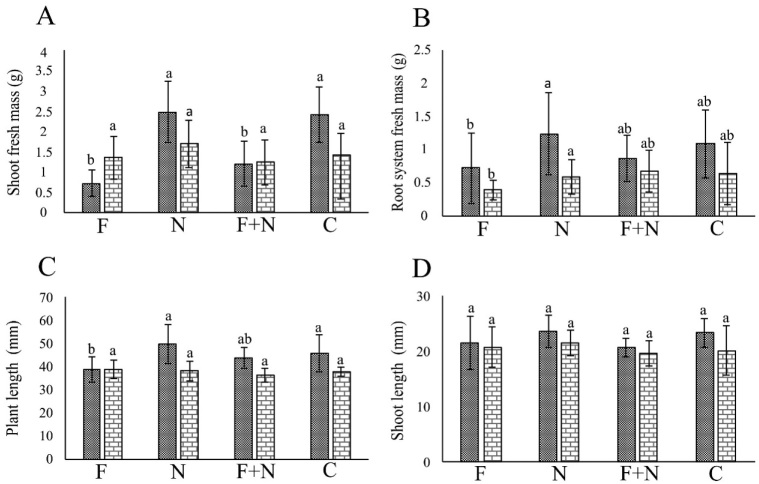
Effect of *Meloidogyne javanica* and *Neocosmospora* sp. 311, acting alone orcombined, on the growth of passion fruit plants cultivated in a growth chamber, in the assays 1 (dotted column) and 2 (brick-pattern column). F: parasitism by fungus only; N: parasitism by nematode only; F + N: parasitism by fungus and nematode; C: noninoculated control. Columns with different letters indicate significant difference between treatments according to the Tukey test at 5%. Values are mean of 10 replicates per treatment for each assay.

In the assays involving *Neocosmospora* sp. 311, the fungus negatively affected (*P* < 0.05) PL, SL, SM and RSM in most assays (Figs. 1,2). *M. incognita* and *M. javanica* did not affect plant growth (*P* > 0.05). Synergy between pathogens occurred only in the reduction of SM in plants co-parasitized by *Neocosmospora* 311 and *M. incognita*, in one assay.

**Figure 3:**
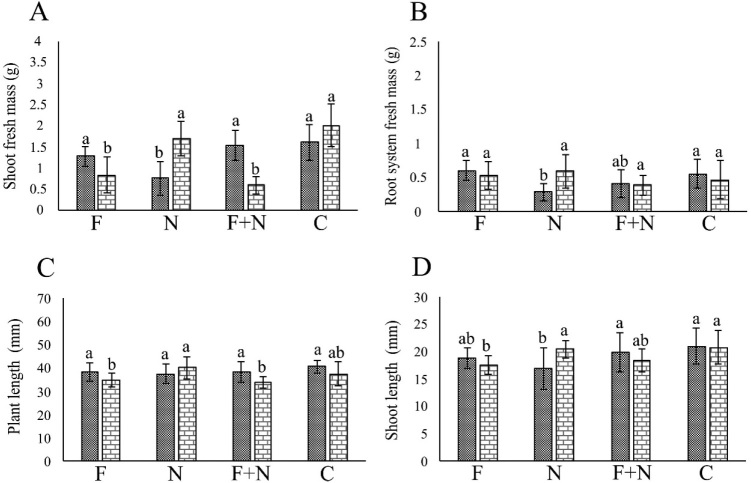
Effect of *Meloidogyne incognita* and *Neocosmospora* sp. 511, acting alone or combined, on the growth of passion fruit plants cultivated in a growth chamber, in assays 1 (dotted column) and 2 (brick-pattern column). F: parasitism by fungus only; N: parasitism by nematode only; F + N: parasitism by fungus and nematode; C: noninoculated control. Columns with different letters indicate significant difference between treatments according to the Tukey test at 5%. Values are mean of 10 replicates per treatment for each assay.

**Figure 4:**
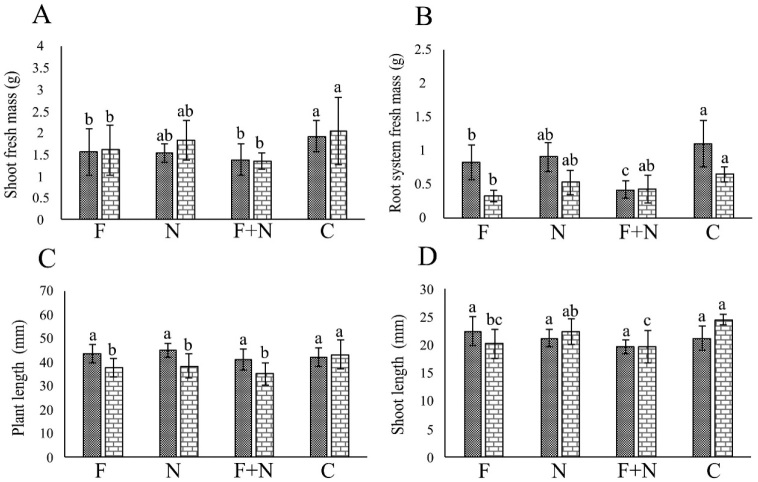
Effect of *Meloidogyne javanica* and *Neocosmospora* sp. 511, acting alone or combined, on the growth of passion fruit plants cultivated in a growth chamber, in assays 1 (dotted column) and 2 (brick-pattern column). F: parasitism by fungus only; N: parasitism by nematode only; F + N: parasitism by fungus and nematode; C: noninoculated control. Columns with different letters indicate significant difference between treatments according to the Tukey test at 5%. Values are mean of 10 replicates per treatment for each assay.

In the assays involving *Neocosmospora* sp. 511, the fungus negatively affected (*P* < 0.05) plant growth in most assays (Figs. 3,4), while *M. incognita* and *M. javanica* did so occasionally. Synergy occurred only in the reduction of RSM in plants co-parasitized by *Neocosmospora* sp. 511 and *M. javanica*, in one assay.

**Figure 5:**
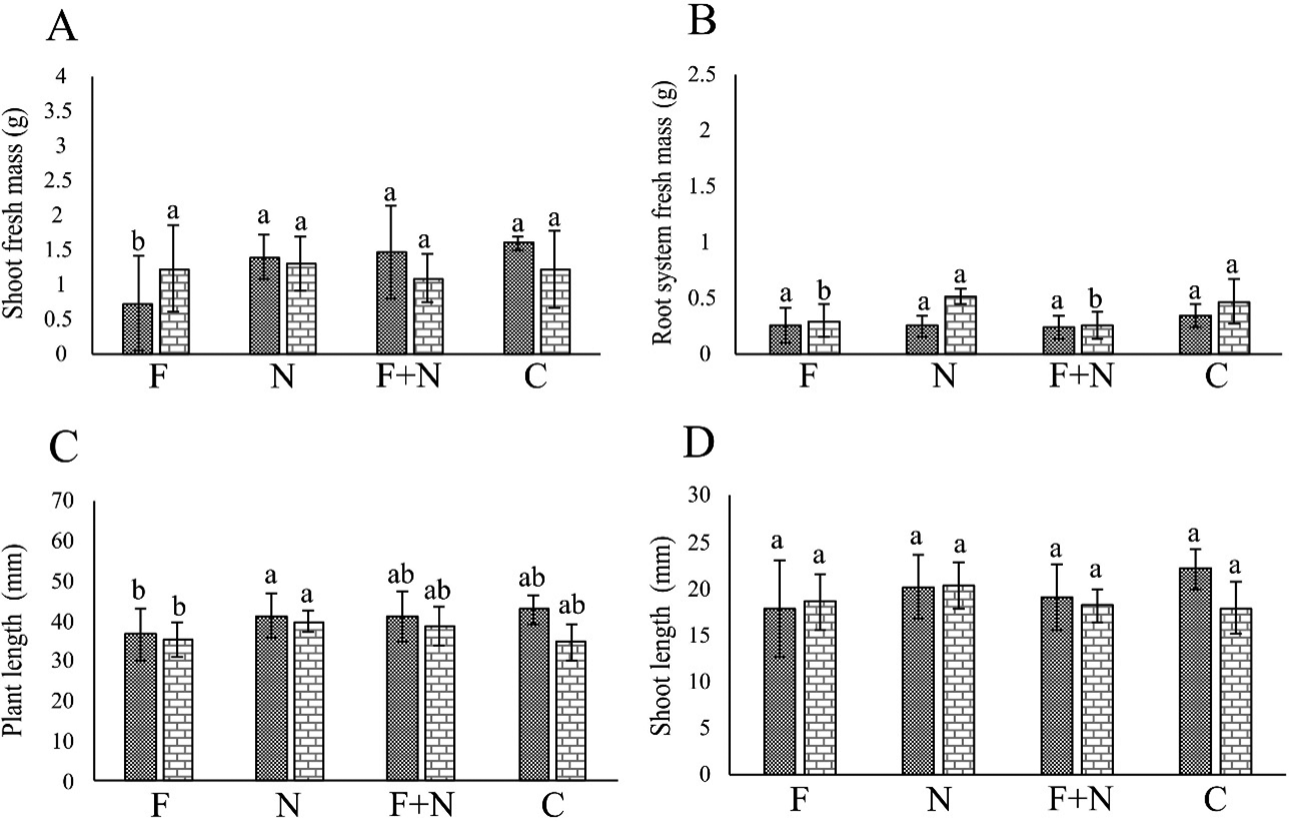
Effect of *Meloidogyne incognita* and *Fusarium nirenbergiae* 022, acting alone or combined, on the growth of passion fruit plants cultivated in a growth chamber, in the assays 1 (dotted column) and 2 (brick-pattern column). F: parasitism by fungus only; N: parasitism by nematode only; F + N: parasitism by fungus and nematode; C: noninoculated control. Columns with different letters indicate significant difference between treatments according to the Tukey test at 5%. Values are mean of 10 replicates per treatment for each assay.

**Figure 6:**
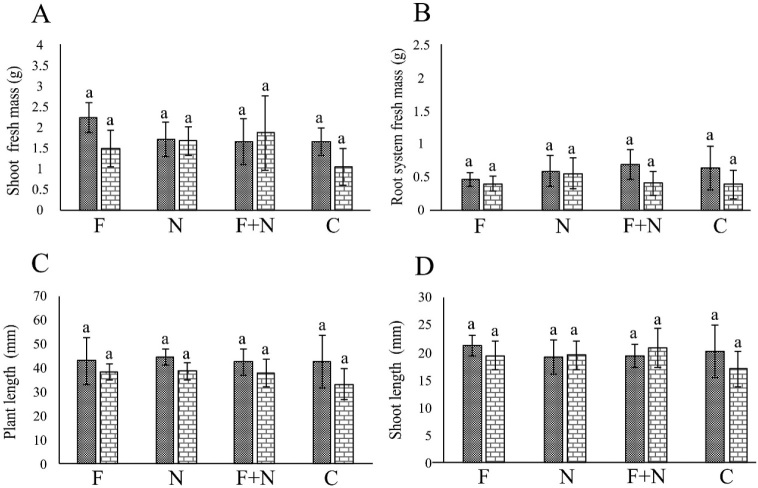
Effect of *Meloidogyne javanica* and *Fusarium nirenbergiae* 022, acting alone or combined, on the growth of passion fruit plants cultivated in a growth chamber, in the assays 1 (dotted column) and 2 (brick-pattern column). F: parasitism by fungus only; N: parasitism by nematode only; F + N: parasitism by fungus and nematode; C: noninoculated control. Columns with different letters indicate significant difference between treatments according to the Tukey test at 5%. Values are mean of 10 replicates per treatment for each assay.

In the assays involving *F. nirenbergiae*, the fungus negatively affected SM and RSM only, in one assay (Figs. 5,6). The nematodes had no effect on plant growth, and no synergy between pathogens occurred.

The nematodes and the fungi, acting alone or combined, had only occasional negative effect (*P* < 0.05) on the plants' RSV (Table 1).

### Lesioned tissue

*Neocosmospora* sp. 311, *Neocosmospora* sp. 511, and *F. nirenbergiae* 022 lesioned the collar-stem region of all plants in all assays. The lesions ascended on the stem up to 86 mm, 38 mm, and 56 mm from soil, respectively. No lesions developed in the nematode-parasitized plants or in the noninoculated control plants. The co-parasitism by *M. incognita* or *M. javanica* had no effect (*P* > 0.05) on the VLT in the collar-stem region (Table 2).

### Severity of shoot symptoms

**Figure S2:**
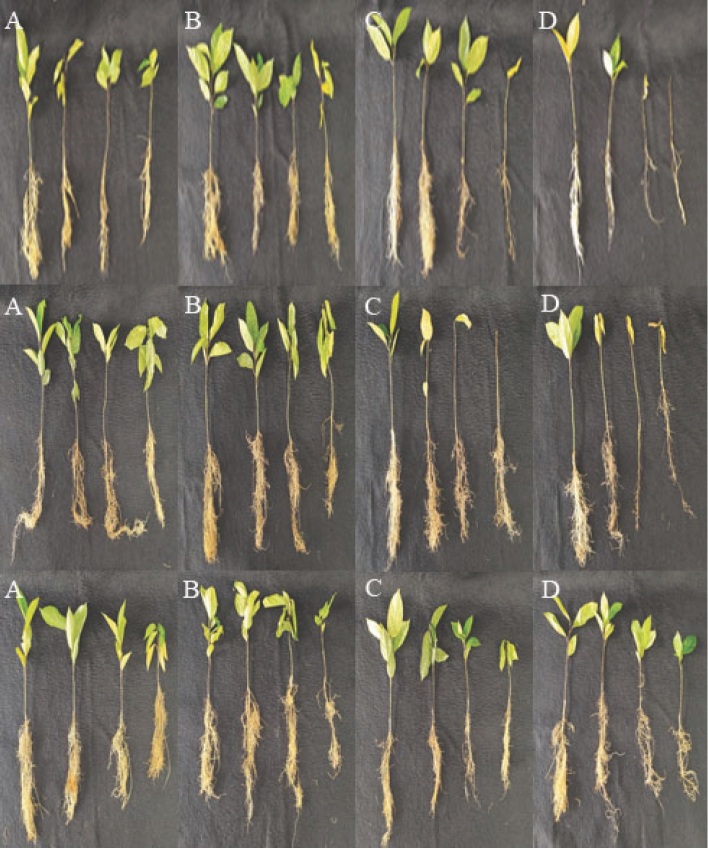
Range of symptoms observed in passion fruit plants in growth chamber assays to investigate the interactions *Meloidogyne incognita*-*Neocosmospora* sp. 311 (top row); *M. incognita*-*Neocosmospora* sp. 511 (middle row); and *M. incognita*-*Fusarium nirenbergiae* F022 (bottom row). (A) non-inoculated control; (B) parasitized by nematode only; (C) parasitized by fungus only; (D) co-parasitized by nematode and fungus.

**Figure S3:**
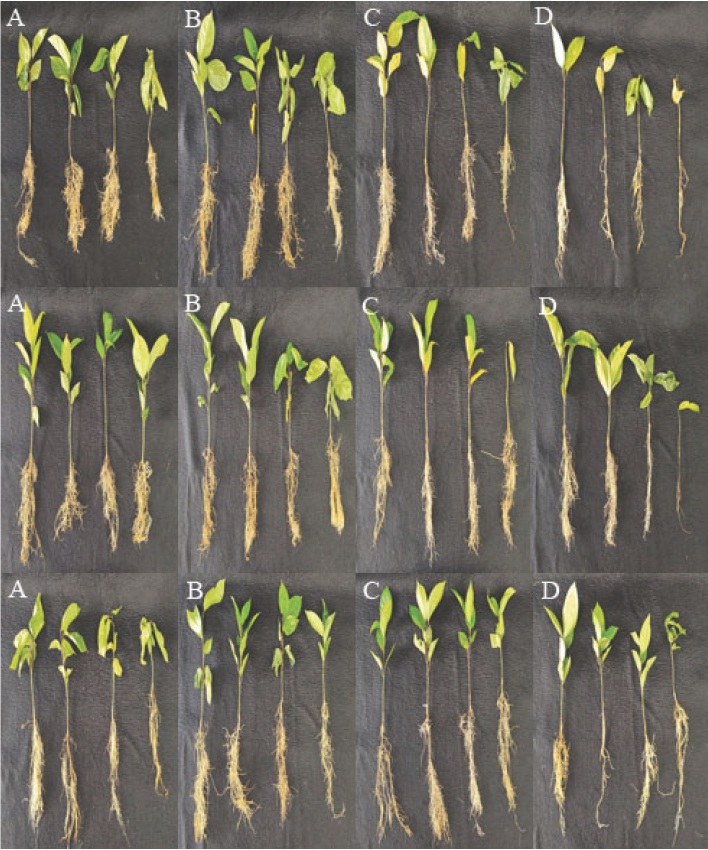
Range of symptoms observed in passion fruit plants in growth chamber assays to investigate the interactions *Meloidogyne javanica*-*Neocosmospora* sp. 311 (top row); *M. javanica*-*Neocosmospora* sp. 511 (middle row); and *M. javanica*-*Fusarium nirenbergiae* F022 (bottom row). (A) non-inoculated control; (B) parasitized by nematode only; (C) parasitized by fungus only; (D) co-parasitized by nematode and fungus.

According to the modified scale of Santos (1997), there were no differences (*P* > 0.05) between the severity of symptoms induced by the nematodes and fungi, alone or combined (Table 3). Nonetheless, visually fungal symptoms were more severe than those caused by nematodes, sometimes leading to plant death (Supplementary Figs. 2,3).

### Nematode reproduction

*M. javanica* and *M. incognita* induced root galls on all inoculated plants, but the maximum RF observed was 0.44 for *M. incognita* and 0.61 for *M. javanica* (Table 4). In the tomato plants used to attest to the viability of the inocula, the mean *M. incognita* RF values were 26 (standard deviation [SD] = ± 14.5) and 9.9 (SD = ± 1.52) in assays 1 and 2, respectively. For *M. javanica*, the values were 48 (SD = ± 27.38) and 8.1 (SD = ± 1.32), respectively. Fungal co-parasitism affected (*P* < 0.05) nematode reproduction positively or negatively in at least one of the assays, but the plants remained resistant to the nematodes (RF < 1).

**Table 4:** Effect of the parasitism by *Neocosmospora* sp. (311 or 511) or *Fusarium nirenbergiae* F022 on the Pf and RF of *Meloidogyne incognita* and *M. javanica* in passion fruit plants cultivated in replicated growth chamber assays (1 and 2).

Treatments	Variables and assays			
	Pf		RF	
	**1**	**2**	**1**	**2**
***M. incognita***	1570 a^a^	677 b	0.44 a	0.19 b
***M. incognita***-***Neocosmospora*** **311**	1180 b	1835 a	0.33 b	0.52 a
	**1**	**2**	**1**	**2**
***M. incognita***	549 a	1070 a	0.15 a	0.3 a
***M. incognita***-***Neocosmospora*** **511**	260 b	1178 a	0.07 b	0.33 a
	**1**	**2**	**1**	**2**
***M. incognita***	210 b	1040 a	0.06 b	0.29 a
***M. incognita***-***F. nirenbergiae*** **F022**	414 a	780 a	0.11 a	0.22 a
	**1**	**2**	**1**	**2**
***M. javanica***	1387 a	1820 a	0.39 a	0.52 a
***M. javanica***-***Neocosmospora*** **311**	445 b	1560 a	0.12 b	0.44a
	**1**	**2**	**1**	**2**
***M. javanica***	465 b	2140 a	0.13 b	0.61 a
***M. javanica***-***Neocosmospora*** **511**	602 a	2260 a	0.17 a	0.64 a
	**1**	**2**	**1**	**2**
***M. javanica***	230 a	1410 a	0.06 a	0.41 a
***M. javanica***-***F. nirenbergiae*** **F022**	155 b	1120 a	0.04 b	0.32 a

a In the column, different letters indicate significant difference between treatments according to the *T* test at 5% significance. Values are means of 10 replicates per treatment for each assay. RF, reproduction factor; Pf, final population.

## Discussion

Our results do not corroborate the anecdotal reports that synergistic interactions occur between root-knot nematodes and *Neocosmospora* sp. or *F. nirenbergiae*. Instead, we observed neutral interactions in all nematode-fungal pairs.

*Neocosmospora* sp. lesioned the plants’ collar-stem region, causing shoot symptoms and impairing growth, while the root-knot nematodes induced relatively mild shoot symptoms but had virtually no impact on plant growth. Co-parasitism caused no additional impact on plant growth, except on SM (isolate 311 + *M. incognita*, in one assay) and RSM (511 + *M. javanica*, in one assay). Co-parasitism had no impact on the extent of lesions caused by the fungus or the plants’ resistance to *M. incognita* and *M. javanica*. *F. nirenbergiae* lesioned the plants’ collar-stem region, caused shoot symptoms, and affected plant growth. No synergy occurred with root-knot nematodes regarding these parameters, and the plant´s resistance to *M. javanica* and *M. incognita* was not affected.

It is important to note that plant resistance to nematodes does not preclude synergistic interactions with soil-borne fungi. For instance, in tomato, chilli, cowpea, white clover, and wheat resistant to nematodes, infection by soil-borne fungi renders them susceptible to the nematode (Hasan, 1985; Hasan and Khan, 1985; Khan and Husain, 1989; Taheri *et al*., 1994; Zahid *et al*., 2002). However, *M. incognita* and *M. javanica* would likely occur in larger numbers in the roots of a susceptible cultivar. The possibility remains that a large nematode root population might have had a greater impact on plant physiology, therefore increasing the possibility of a disease complex occurring with soil-borne fungi. The resistance of “UENF Rio Dourado” to both root-knot nematodes was a serendipitous finding of this study. This cultivar was registered recently (Viana *et al*., 2016) with no information on nematode resistance. It is noteworthy that the root-knot nematodes induced shoot symptoms despite their poor reproduction. Presumably, passion fruit resistance to root-knot nematodes is post-infectional and metabolically costly, as reported for many crops. Hence, in nematode-infested fields, it seems advisable to reduce soil nematode density prior to establishing a passion fruit orchard, even if a resistant cultivar is to be planted.

In the field, passion fruit plants may be subjected to prolonged water deficits and/or suboptimal soil conditions involving physical and fertility aspects. Such abiotic stresses might interact with both nematodes and soil-borne fungi and give rise to a disease complex. Field experiments aimed at investigating these abiotic and biotic interactions would be challenging due to the perennial nature of the crop and the uneven severity of nematode and fungal symptoms in large experimental plots.

In our assays, the seedlings presented a relatively slow initial development, with few roots in the first weeks. This prompted us to delay nematode inoculation to 60 DAS. The fungal inoculation method of Fisher *et al*. (2010) provided consistent plant infection and symptoms, allowing inference on pathogen interactions. Deeping seedlings´ roots in a suspension of fungal spores often results in uneven infection, which precluded inferences by Rocha *et al*. (2021). *Neocosmospora* sp. and *F. nirenbergiae* cause quick and severe decay of the collar-stem region on passion fruit plants. Fungal inoculation prior to or synchronous with nematode inoculation would preclude nematode development and inference on pathogen interactions. We used a longer lapse between inoculations of nematodes and fungi than Fischer *et al*. (2010) – 30 d – because we presumed that the slow initial development of passion fruit plants in the growth chamber might slow nematode development. A shorter lapse between inoculations might reveal a synergistic interaction between the pathogens, particularly in cultivars highly susceptible to nematodes.

The susceptibility of passion fruit plants to *Neocosmospora* sp. and *F. nirenbergiae* and the damage caused by root-knot nematodes suggest that breeders should attempt to combine resistance to these pathogens in new cultivars and rootstocks, even if no synergy occurs between them. The widespread incidence of these pathogens suggests that multi-resistant genotypes would likely perform better in fields. To the best our knowledge, no passion fruit breeding program has attempted this, and genotype screenings continue to be conducted for single pathogens only (e.g., Preisigke *et al*., 2015; Nascimento *et al*., 2016; Zucareli *et al*., 2020; Carvalho *et al*., 2021).

## Figures and Tables

**Table S1:** . *P* values obtained in the Shapiro–Wilk test at 5% for the effect of pathogens, acting alone or combined, on passion fruit plants cultivated in replicated growth chamber assays (1 and 2).

Assays		Variables and interactions			
	SM	RSM	PL	SL	RSV
		***Meloidogyne incognita -Neocosmopora* sp. 311**			
**1**	0.21	0.34	0.08	0.07	6.53 × 10^–9^
**2**	0.37	0.17	0.63	0.52	0.005
		***M. incognita Neocosmopora* sp. 511**			
**1**	0.41	0.31	0.07	0.1	5.61 × 10^–6^
**2**	0.69	0.26	0.74	0.07	3.74 × 10^–12^
		***M. incognita Fusarium nirenbergiae*** F022			
**1**	0.27	0.46	0.2	0.71	7.74 × 10^–6^
**2**	0.76	0.52	0.81	0.24	5.21 × 10^–6^
		***Meloidogyne javanica*** -*Neocosmopora* **sp. 311**			
**1**	0.98	0.11	0.33	0.55	0.0001
**2**	0.99	0.17	0.79	0.33	1.84 × 10^–12^
		***M. javanica*****-** ***Neocosmopora*** **sp. 511**			
**1**	0.08	0.51	0.31	0.4	0.02
**2**	0.14	0.92	0.57	0.64	4.26 × 10^–8^
		***M. javanica*** **-*F. nirenbergiae*** **F022**			
**1**	0.09	0.86	0.06	0.39	0.046
**2**	0.63	0.4	0.63	0.38	1.84 × 10^–12^

SM, shoot fresh mass; RSM, root system fresh mass; PL, plant length; SL, shoot length; RSV, root system volume.

**Table S2:** . *P* values obtained in the Shapiro–Wilk test at 5% for the VLT in the collar-stem region and LL caused by *Neocosmospora* sp. (311 or 511) or *Fusarium nirenbergiae* F022, and for the Pf and RF of *Meloidogyne incognita* and *M. javanica*, in passion fruit plants cultivated in replicated growth chamber assays (1 and 2).

Assays		Variables and interactions		
	VLT	LL	Pf	RF
		***M. incognita*** **-*Neocosmopora* sp. 311**		
**1**	0.12	0.28	0.08	0.08
**2**	0.07	0.16	1.74 × 10^–5^	1.74 × 10^–5^
		***M. incognita*** **-*Neocosmopora* sp. 511**		
**1**	0.24	0.42	0.02	0.02
**2**	0.79	0.09	0.71	0.71
		***M. incognita*** **-*F. nirenbergiae* F022**		
**1**	0.48	0.58	0.03	0.03
**2**	0.38	0.89	0.06	0.06
		***M. javanica*** **-*Neocosmopora* sp. 311**		
**1**	0.36	0.59	0.002	0.002
**2**	0.13	0.35	0.45	0.45
		***M. javanica*** **-*Neocosmopora* sp. 511**		
**1**	0.15	0.14	0.02	0.02
**2**	0.37	0.18	0.81	0.81
		***M. javanica*** **-*F. nirenbergiae* F022**		
**1**	0.95	0.00063	0.004	0.004
**2**	0.12	0.0047	0.15	0.15

LL, length of the lesion; RF, reproduction factor; VLT, volume of lesioned tissue; Pf, final population.

**Table S3:** Mean squares obtained in ANOVA for the effect of pathogens, acting alone or combined, on passion fruit plants cultivated in replicated growth chamber assays.

Parameters	Variables and interactions			
	SM	RSM	PL	SL
	***Meloidogyne*** ***incognita*****-** ***Neocosmospora*** **sp. 311**			
Assay	1.53*	0.25	288.9**	1.31
Treatment	5.9**	0.39**	209.98**	62.61**
Assay × treatment	1.03**	0.96**	19.49	18.22
Residual	0.25	0.09	19.88	9.78
CV%	44.7	54.6	12.13	17.19
	***M***. ***incognita*****-*Neocosmospora*** **sp. 511**			
Assay	0.002	0.02	33.61	0**
Treatment	2.5**	0.11*	68.53**	30.78**
Assay × treatment	3.6**	0.17**	47.59*	32.26**
Residual	0.14	0.04	12.36	6.22
CV%	28.89	42.23	9.41	12.9
	***M***. ***incognita*****-*Fusarium*** ***nirenbergiae*** **F022**			
Assay	0.16	0.24**	224.79**	24.16
Treatment	0.74*	0.18**	85.4*	19.32
Assay × treatment	0.86*	0.09**	54.07	24.68
Residual	0.24	0.01	24.83	10.19
CV%	39.45	35.12	12.91	16.62
	***Meloidogyne*** ***javanica*****-** ***Neocosmospora*** **sp. 311**			
Assay	1.61*	3.27**	913.95**	62.36*
Treatment	5.27**	0.5*	100.27*	20.24
Assay × treatment	2.86**	0.19	122.88**	5.83
Residual	0.35	0.18	29.01	10.72
CV%	39.13	54.9	13.11	15.29
	***M***. ***javanica*****-*Neocosmospora*** **sp. 511**			
Assay	0.22	2.21**	377.41**	5.84
Treatment	1.32**	0.74**	72.12*	33.86**
Assay × treatment	0.11	0.28**	66.3*	27.16**
Residual	0.22	0.04	19.35	4.53
CV%	28.39	32.09	10.80	9.93
	***M***. ***javanica*****-*F. nirenbergiae*** **F022**			
Assay	1.8**	0.48**	1207.61**	13.28
Treatment	0.98*	0.08	16.35	12.57
Assay × treatment	1.07**	0.08	85.72*	21.38
Residual	0.26	0.05	27.59	9.59
CV%	30.45	41.85	12.82	15.79

“**” and “*” indicate significant variance according to the *F* test at 1% and 5% significance, respectively. SM, shoot fresh mass; RSM, root system fresh mass; PL, plant length; SL, shoot length.
